# Application of dynamic optimisation for planning a haemodialysis process

**DOI:** 10.1186/s12882-019-1409-8

**Published:** 2019-07-02

**Authors:** Wojciech Stecz, Radoslaw Pytlak, Aleksandra Rymarz, Stanislaw Niemczyk

**Affiliations:** 1 Military University of Technology, Faculty of Cybernetics, Kaliskiego 2, Warsaw, 00-908 Poland; 2 Warsaw University of Technology, Faculty of Mathematics and Information Science, Koszykowa 75, Warsaw, 00-662 Poland; 3 Military Institute of Medicine, Szaserow 128, Warsaw, 04-141 Poland

**Keywords:** Haemodialysis planning, Dynamic optimisation, IV-compartment model

## Abstract

**Background:**

The aim of the study is to show that optimization tools can be used in planning the haemodialysis process in order to obtain the most effective treatment aimed at removing both urea and phosphorus. To this end we use the IV–compartment model of phosphorus kinetics.

**Methods:**

The use of the IV–compartment model of phosphorus kinetics forces us to apply new numerical tools which cope with a rebound phenomenon that can occur during haemodialysis. The proposed algorithm solves optimization problems with various constraints imposed on concentrations of urea and phosphorus.

**Results:**

We show that the optimization tools are effective in planning haemodialysis processes aimed at achieving desired levels of urea and phosphorus concentrations at the end of these processes. One of the numerical experiments reported in the paper concerns patients data who experienced a rebound phenomenon during haemodialysis due to a low level of phosphorus concentration.

**Conclusion:**

In order to plan haemodialysis processes one should take into account the fact that these processes, in general, are described by different equations in different regions determined by phosphorus concentrations. This follows from the fact that mechanisms modelled by IV–compartment model are activated during dialysis. Therefore, advanced numerical tools have to be used in order to simulate and optimize these processes. The paper shows that these tools can be constructed and effectively applied in planning haemodialysis processes.

## Background

Hiperphosphatemia is associated with increased mortality among dialysis patients primarily due to accelerated cardiovascular disease (CVD) [1, 2]. Altered calcium, phosphorus and PTH levels which accompany chronic kidney disease, as well as an excessive burden of calcium supplied by calcium-based oral phosphorus binders are responsible for exaggerated vascular calcification and lead to enhanced atherosclerosis in this population [3].

European guidelines recommend lowering the phosphorus level in the blood towards the normal range [4]. Serum phosphorus concentration in haemodialysis patients derives from phosphorus content in the diet, its elimination with residual renal function and its removal during haemodialysis (HD) sessions. Therefore, therapeutic approaches focus on two directions: the usage of oral binders which decreases phosphorus absorption from the intestinum, as well as effective phosphorus elimination by different modes of haemodialysis treatment. The problem for establishing the most efficient schedule of haemodialysis treatment is that the kinetics of phosphorus is much more complicated than other molecules such as urea or creatinine. During the first hour of an haemodialysis session, phosphorus concentration rapidly decreases, followed by a relatively stable level (plateau) until the end of haemodialysis. After haemodialysis the phosphorus level increases rapidly reaching the predialytic level within 4–8 h [5]. Therefore the most effective strategy is to extend weekly dialysis time by enhancing the length of the sessions and/or by increasing the frequency of the sessions. It is estimated that the weekly dialysis time required to avoid the usage of oral binders is 18–30 h. Other methods such as increasing blood flow rate, greater dialyzer size, increased dialysate flow rate are also used to increase phosphorus elimination however they are less effective [6].

Many mathematical models, from one to multi–compartment models, have been applied to describe phosphorus kinetics ([7–11]). During haemodialysis session phosphorus is removed from the plasma which is a compartment accessible for the treatment mode. The intradialytic plateau of phosphorus level suggests the existence of second phosphorus storage which is inaccessible for the dialyser and provides a continuous inflow of phosphorus resulting in the plateau of its level in the second part of haemodialysis. Therefore a two–pool model was proposed. However, it did not completely explain the constant phosphorus level during haemodialysis. Therefore Spalding et al. proposed a four-compartment model which suggested gradual phosphorus mobilization from the pools during dialysis [11]. Gotch et al. proposed intracellular volume as a site of phosphorus storage [12]. Another conception of phosphorus kinetics assumes a pseudo I–compartment model where the phosphorus level during haemodialysis is determined by its clearance from distal compartment. The problem is that the rate of this clearance is dependent on the patient and clinical circumstances [13].

The aim of the study was to introduce advanced numerical tools in planning the haemodialysis process in order to obtain the most effective treatment in removing urea and phosphorus as well. The mathematical model assumes the modified IV–compartment model of phosphorus kinetics.

The organisation of the paper is as follows. “Methods” section gives the introduction to the haemodialysis problem. We concentrate on the mathematical models for urea and phosphorus kinetics. Next we present an optimisation procedure that can be used for haemodialysis. In “Results” section we present some simulation and optimisation experiments. Last section discusses our results and possible future research directions.

## Methods

***Models of urea kinetics***

**Fig. 1:**
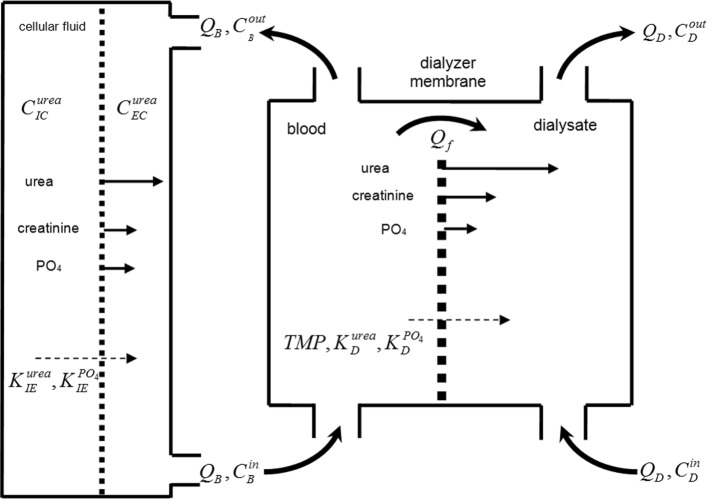
II-compartment kinetic model of toxins and substances

The II–compartment model depicts a patient as a two–compartment system of different volumes of solutes that are regularly cleared, i.e., intracellular fluid and extracellular fluid. Between these solutes, diffusion of substances takes place according to different substances’ concentrations. II–compartment model is shown in Fig. 1.

According to [11, 14] the equations describing II–compartment model are as follows:

1 $$ {\begin{aligned} \frac{{dC}_{EC}^{urea}}{dt} & = \frac{K_{IE}^{urea} \cdot \left(C_{IC}^{urea}-C_{EC}^{urea}\right) - C_{EC}^{urea} \cdot \left(K_{D}^{urea}+K_{r}^{urea}+K^{ufr}\right) }{0.34 \cdot V(0)-UFR} \end{aligned}}  $$

2 $$ {\begin{aligned} \frac{{dC}_{IC}^{urea}}{dt} & = \frac{K_{IE}^{urea}\cdot\left(C_{EC}^{urea}-C_{IC}^{urea}\right)+G^{urea}}{0.66 \cdot V(0)} \end{aligned}}  $$

3 $$ {\begin{aligned} \frac{dUFR}{dt} & = U_{ufr} \end{aligned}}  $$

where:

*UFR* - ultrafiltration volume

*U*_*ufr*_ - profile of the ultrafiltration rate

*K*^*ufr*^ - ultrafiltration coefficient

$K_{r}^{urea}$ - urea clearance associated with residual renal function

$K_{D}^{urea}$ - dialyser clearance

$\frac {{dC}_{EC}^{urea}}{dt}$ - variation of urea concentration in the extracellular fluid

$\frac {{dC}_{IC}^{urea}}{dt}$ - variation of urea concentration in the intracellular fluid

$K_{IE}^{urea}$ - diffusion speed between inner and outer cellular solute through a cellular membrane - the same in both directions

*G*^*urea*^ - urea generation in the human cells according to quick variations of ions during haemodialysis - unsurveyed process, but was observed - we set *G*^*urea*^ to zero in our simulation and optimisation tests.

In the above equations we use the Watson formula ([15]) of intracellular to extracellular volume ratio of 2:1 (0.66·*V*(0) for intracellular volume and 0.34·*V*(0) for extracellular volume).

***Models of phosphorus kinetics***

**Fig. 2:**
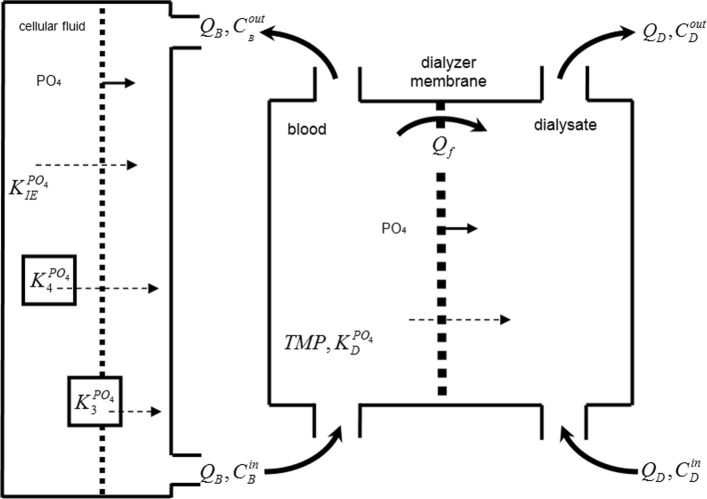
IV-compartment kinetic model of toxins and substances

In the paper we rely on the models described in the papers ([7, 11, 16]) presenting the phosphorus kinetics. We use especially Spalding’s IV–compartment model shown in Fig. 2.

As far as phosphorus kinetics is concerned we have employed the model presented in ([7, 11]):

4 $$\begin{array}{*{20}l} \frac{{dC}_{EC}^{{PO}_{4}}}{dt} & = & \frac{K_{IE}^{{PO}_{4}} \cdot (C_{IC}^{{PO}_{4}}-C_{EC}^{{PO}_{4}}) - K_{D}^{{PO}_{4}} \cdot C_{EC}^{{PO}_{4}}}{0.34 \cdot V(0)-UFR} +  \end{array} $$

5 $$\begin{array}{*{20}l} && K_{3}^{{PO}_{4}} + K_{4}^{{PO}_{4}}  \\ \frac{{dC}_{IC}^{{PO}_{4}}}{dt} & = & \frac{K_{IE}^{{PO}_{4}} \cdot (C_{EC}^{{PO}_{4}}-C_{IC}^{{PO}_{4}}) }{0.66 \cdot V(0)}  \end{array} $$

where:

$\frac {{dC}_{EC}^{{PO}_{4}}}{dt}$ - variation of phosphorus concentration in the extracellular fluid

$\frac {{dC}_{IC}^{{PO}_{4}}}{dt}$ - variation of phosphorus concentration in the intracellular fluid

$K_{IE}^{{PO}_{4}}$ - diffusion speed between inner and outer cellular solute through a cellular membrane - the same in both directions

$K_{D}^{{PO}_{4}}$ - phosphorus clearance coefficient in dialyzer

$K_{3}^{{PO}_{4}}$ - phosphorus generation coefficient - used after exceeding the upper boundary of phosphorus concentration for a healthy person

$K_{4}^{{PO}_{4}}$ - phosphorus generation coefficient - used after exceeding the lower boundary of phosphorus concentration for a healthy person

Additionally, on the basis of the analysis given in [11], we model the qualitative change in equations (4)–(5) by the following algebraic equations (thereby, among other things, functions stated in [11] are continuous):

6 $$\begin{array}{*{20}l} K_{4}^{{PO}_{4}} &=& \alpha \cdot \max \left (C_{min}^{{PO}_{4}}-C_{IC}^{{PO}_{4}},0\right) \end{array} $$

7 $$\begin{array}{*{20}l} K_{3}^{{PO}_{4}} &=& \beta \cdot \max \left (C_{max}^{{PO}_{4}}-C_{IC}^{{PO}_{4}},0\right)  \end{array} $$

where:

*α*,*β* - coefficients that describe the speed of returning the phosphorus concentrations to the initial levels. $K_{3}^{{PO}_{4}}$ is activated only when a phosphorus level is below $C_{max}^{{PO}_{4}}$ value (third compartment). And $K_{4}^{{PO}_{4}}$ is activated only when a phosphorus level is below $C_{min}^{{PO}_{4}}$ respectively (fourth compartment).

Furthermore, we underline that a phosphorus clearance coefficient $K_{D}^{{PO}_{4}}$ is a function of two parameters: predefined by producer phosphorus clearance of the dialyser membrane $K_{D,base}^{{PO}_{4}}$, and the clearance reduction coefficient *κ* for the patient (in literature, one may see that from some group of patients there are suggested values of *κ* [7, 17], we assume value *κ*=0.6 for hollow fiber dialyzers we used).

8 $$ K_{D}^{{PO}_{4}} = \kappa \cdot K_{D,base}^{{PO}_{4}}.   $$

To check our model we used data of two patients such as: age, gender, body mass, height, serum urea and phosphorus levels, the dialyzers size, value of blood and dialysate flow during dialysis session, ultrafiltration volume. On the basis of the data models parameters were determined for each patient by simulating equations (1)–(8) (see Table 1). Hollow fiber dialyzers made by Allmed (Polypure series) or Gambro (Polyflux series) were used. The study protocol was accepted by the local ethical committee. Each participant signed the informed consent.

**Table 1:** Parameters for simulation of haemodialysis process

First patient (71 y.o. male)				
*t*_*D*_=240[min]				
*Q*_*B*_=280[ml/min], *Q*_*D*_=500[ml/min], *U*_*ufr*_=4.08[ml/min], *α*=0.05, *β*=300.0, *κ*=0.65				
$C^{{PO}_{4}}_{min}=0.70 \text {[mmol/L]}$, $C^{{PO}_{4}}_{max}=0.85 \text {[mmol/L]}$				
*K*_0_*A*=600 [ml/min], *K*^*ufr*^=12.5 [ml/(min·mmHg)], $K_{r}^{urea}=0\ \text {[ml/min]},K_{D}^{{PO}_{4}}=220\ \text {[ml/min]}$				
$K_{IE}^{urea}=750.0 \text {[ml/min]}$, $K_{IE}^{{PO}_{4}}=350 \text {[ml/min]}$, *G*^*urea*^=0.0[mmol/min], *V*(0)=48430[ml]				
Input data - first patient parameters				
$C_{EC}^{{PO}_{4}}(0)$	$C_{IC}^{{PO}_{4}}(0)$	$C_{EC}^{urea}(0)$	$C_{IC}^{urea}(0)$	*UFR*(0)
[mmol/L]	[mmol/L]	[mmol/L]	[mmol/L]	[ml]
1.776	1.776	16.881	16.881	0.0
Second patient (55 y.o. male)				
*t*_*D*_=240[min]				
*Q*_*B*_=280[ml/min], *Q*_*D*_=500[ml/min], *U*_*ufr*_=11.25[ml/min], *α*=0.05, *β*=300.0, *κ*=0.65				
$C^{{PO}_{4}}_{min}=0.70 \text {[mmol/L]}$, $C^{{PO}_{4}}_{max}=0.85 \text {[mmol/L]}$				
*K*_0_*A*=600[ml/min], *K*^*ufr*^=68.0[ml/(min·mmHg)], $K_{r}^{urea}=0$$\text {[ml/min]},K_{D}^{{PO}_{4}}=220 \text {[ml/min]}$				
$K_{IE}^{urea}=750.0 \text {[ml/min]}$, $K_{IE}^{{PO}_{4}}=350 \text {[ml/min]}$, *G*^*urea*^=0.0[mmol/min], *V*(0)=43440[ml]				
Input data - second patient parameters				
$C_{EC}^{{PO}_{4}}(0)$	$C_{IC}^{{PO}_{4}}(0)$	$C_{EC}^{urea}(0)$	$C_{IC}^{urea}(0)$	*UFR*(0)
[mmol/L]	[mmol/L]	[mmol/L]	[mmol/L]	[ml]
0.872	0.872	18.536	18.536	0.0

***Haemodialysis optimisation problem***

The main optimisation problem considered in the paper is stated as follows. Having combined kinetic models of urea and phosphorus we look for proper concentrations of urea and phosphorus and the end of the haemodialysis process by controlling the parameters *Q*_*B*_, *Q*_*D*_, *U*_*ufr*_. In other words, by solving the optimal control problem we want to choose a proper dialysis membrane in order to achieve final parameters of haemodialysis.

That optimisation problem can be formulated as follows:

9 $$ \min_{Q_{B},Q_{D},U_{ufr}}C_{EC}^{urea}(t_{D})   $$

subject to the constraints (1)–(8), the following constraints at final time *t*_*D*_

10 $$\begin{array}{*{20}l} & C_{EC}^{urea}(t_{D}) &\leq L^{urea}_{EC}  \end{array} $$

11 $$\begin{array}{*{20}l} & C_{IC}^{urea}(t_{D}) &\leq L^{urea}_{IC}  \end{array} $$

12 $$\begin{array}{*{20}l} L_{min}^{UFR} \leq & UFR(t_{D}) &\leq L_{max}^{UFR},  \end{array} $$

and the constraints on the control variables

13 $$\begin{array}{*{20}l} Q_{B}^{min} \leq & Q_{B}(t) &\leq Q_{B}^{max}  \end{array} $$

14 $$\begin{array}{*{20}l} Q_{D}^{min} \leq & Q_{D}(t) &\leq Q_{D}^{max}  \end{array} $$

15 $$\begin{array}{*{20}l} U_{ufr}^{min} \leq & U_{ufr}(t) &\leq U_{ufr}^{max},\ t\in [0,t_{D}],  \end{array} $$

where $L^{urea}_{EC}$ and $L^{urea}_{IC}$ mean maximum admissible values for urea concentrations. $L_{min}^{UFR}$ and $L_{max}^{UFR}$ mean minimum and maximum admissible values for the ultrafiltration. $Q_{B}^{min}$, $Q_{B}^{max}$, $Q_{D}^{min}$, $Q_{D}^{max}$, $U_{ufr}^{min}$, $U_{ufr}^{max}$ define bound constraints on decision variables, in particular $U_{ufr}^{min}$, $U_{ufr}^{max}$ limit the rate of ultrafiltration.

The stated optimisation problem is difficult to solve since it is defined by hybrid differential equations ([18, 19]). Hybrid systems are described by both discrete and continuous variables—discrete variables indicate regions in which unique systems equations are applied, continuous variables are solutions to these equations. The model of haemodialysis has three discrete states which are defined by the phosphorus concentration threshold values $C_{min}^{{PO}_{4}}$ and $C_{max}^{{PO}_{4}}$: if $C_{IC}^{{PO}_{4}}$ has lower value than $C_{min}^{{PO}_{4}}$ then the process is in the first discrete state; if $C_{IC}^{{PO}_{4}}$ is greater, or equal to $C_{min}^{{PO}_{4}}$ but has lower value than $C_{max}^{{PO}_{4}}$ then we say that the process is in the second discrete state; eventually when $C_{IC}^{{PO}_{4}}$ assumes the values greater than $C_{max}^{{PO}_{4}}$, the system is in the third discrete state. In each discrete state, the haemodialysis process is described by a different set of differential equations. The switch from one discrete state to another is trigged when one of the switching conditions is satisfied: $C_{IC}^{{PO}_{4}}(t_{s})=C_{min}^{{PO}_{4}}$, or $C_{IC}^{{PO}_{4}}(t_{s})=C_{max}^{{PO}_{4}}$, for some switching time *t*_*s*_.

The numerical procedure which we used to solve the problem (9), (1)–(8),(10)–(15) is described, to much extent, in [20] and [21]. The main features of the procedure are:

- it is based on the Radau IIa version of a Runge–Kutta method for integrating differential equations,
- it uses adjoint equations to evaluate gradients of functions defining the optimization problem.

As far as the point a) is concerned we use a Runge–Kutta method in our optimisation procedure since we are dealing with optimal control problems and for these problems these integration procedures are the most suitable (the justification of that claim is given in Chapter 6 of [22]). Our optimisation method is based on the RADAU5 procedure ([23]) which we had to modify by incorporating into it subroutines for the location of switching points *t*_*s*_.

Furthermore, we had to add to the RADAU5 procedure the subroutine for the consistent evaluation of adjoint equations associated with the equations which are used by the RADAU5 procedure for the evaluation of system equations. The second modification was needed in order to realize the feature b) of our method. In our opinion the optimisation method for solving optimal ontrol problems with hybrid systems should follow the scheme in which at every optimisation procedure step system equations are solved first (due to the necessity of the switching points location) and then values and gradients of optimization functions are calculated. If we pursue the scheme we are in fact limited to the use of adjoint equations in gradient evaluations (more on that issue is in [20]).

The overview of our optimisation algorithm is as follows:

1. Set initial values of the controls *u*_0_ (*u*=(*Q*_*B*_,*Q*_*D*_,*U*_*ufr*_)) and *k*=0.
2. Integrate hybrid system equations for given initial conditions and the current controls *u*_*k*_, while integrating system equations locate the switching points $\{{t_{s}^{l}}\}$. Calculate values of all functionals which define the optimisation problem.
3. Evaluate adjoint equations for each optimisation problem’s functional. Having trajectories of adjoint equations evaluate gradients of the problem functionals.
4. On the basis of calculated gradients determine whether current controls *u*_*k*_ satisfy necessary optimality conditions. If this is the case then STOP, otherwise find new controls *u*_*k*+1_ (using some algorithm which refers to the gradients), increase *k* by one and go to Step 2.

## Results

***Simulation and optimisation results***

Both simulation and optimization were performed on the data of two patients. We first performed simulation of the model stated in the previous sections to verify its correctness and to analyse the relative behaviour of trajectories $C^{urea}_{EC}$, $C^{urea}_{IC}$, $C^{{PO}_{4}}_{EC}$ and $C^{{PO}_{4}}_{IC}$.

The coefficients used in simulation are given in Table 1. Notice that we assumed the following constant values for the control variables: *Q*_*D*_=500[ml/min] and *Q*_*B*_=280[ml/min]. A constant ultrafiltration rate (*U*_*ufr*_=4.08[ml/min] for a first patient, *U*_*ufr*_=11.25[ml/min] for a second patient) was assumed throughout haemodialysis. These values, together with the other haemodialysis parameters, may be considered as typical.

**Fig. 3:**
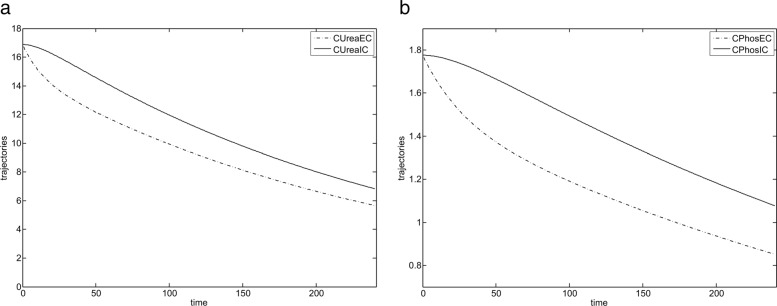
Simulation results for IV-compartment model (first patient). **a** IV-compartment model presenting variations of urea concentration during haemodialysis. **b** IV-compartment model presenting variations of phosphorus concentration during haemodialysis

Haemodialysis process simulation was conducted in OpenModelica (which implements Modelica standard [24] for simulation) using integration method dassl, with tolerance 1e-6, simulation period was set to 240 time units. Exemplary simulation results of variations of an urea level and a phosphorus level in plasma for the presented equations of IV–compartment system are shown in Fig. 3. They show that while lowering phosphorus level we at the same time also decrease urea quantities. That relative behaviour of urea and phosphorus concentrations justifies our optimisation problem in which we only minimize the urea concentration at the end of the haemodialysis process (in that way we avoid solving more difficult multicriteria optimisation problem).

In the optimisation problem we used the model with the same coefficients as in the simulation experiment with the exception that control variables *Q*_*D*_, *Q*_*B*_, *U*_*ufr*_ were not fixed but determined by the optimisation procedure outlined in the previous section.

For the first patient three optimization problems were solved for different constraints imposed on control variables – we changed the optimisation parameters such as $Q_{D}^{min}$, $Q_{D}^{max}$, $Q_{B}^{min}$ and $Q_{B}^{max}$. In the first run we set $Q_{D}^{max}=550$ [ml/min] and $Q_{B}^{max}=300$ [ml/min], in the second run $Q_{D}^{max}=650$ [ml/min] and $Q_{B}^{max}=400$ [ml/min]. In the second run we increased the control parameters $Q_{D}^{max}$ and $Q_{B}^{max}$ to achieve assumed final concentrations of urea.

We have observed that for the data of both patients, in order to minimise the objective function, the solver set the variable values (*Q*_*B*_ and *Q*_*D*_) to the maximum permissible values. It becomes obvious if we notice that we minimise the concentration of urea, so increasing the flow of fluids (blood and dialysate) is necessary.

However, the above conclusion is no longer valid when we impose additional constraints, for example on the value of $C_{IC}^{{PO}_{4}}$ at final time *t*_*D*_ – in that case in addition to constraints (10)–(15) we impose the constraint (that case is stated in Table 2 as Run no. 3)

16 $$\begin{array}{@{}rcl@{}} L_{IC}^{PO} & \leq & C_{IC}^{{PO}_{4}}(t_{D}). \end{array} $$

**Table 2:** Parameters for optimisation of haemodialysis process

First patient 71 y.o. male			
*t*_*D*_=240[min]			
Run no. 1: $L^{urea}_{EC}=6.0 \text {[mmol/L]}$, $L^{urea}_{IC}=7.0 \text {[mmol/L]}$			
Run no. 2: $L^{urea}_{EC}=5.0 \text {[mmol/L]}$, $L^{urea}_{IC}=6.0 \text {[mmol/L]}$			
Run no. 3: $L^{urea}_{EC}=6.0 \text {[mmol/L]}$, $L^{urea}_{IC}=7.0 \text {[mmol/L]}$, $L_{IC}^{PO} = 0.85 \text {[mmol/L]}$			
Run no. 1: $Q_{B}^{min}=250 \text {[ml/min]}$, $Q_{B}^{max}=300 \text {[ml/min]}$, $Q_{D}^{min}=400 \text {[ml/min]}$, $Q_{D}^{max}=550 \text {[ml/min]}$			
Run no. 2: $Q_{B}^{min}=250 \text {[ml/min]}$, $Q_{B}^{max}=400 \text {[ml/min]}$, $Q_{D}^{min}=400 \text {[ml/min]}$, $Q_{D}^{max}=650 \text {[ml/min]}$			
Run no. 3: $Q_{B}^{min}=250 \text {[ml/min]}$, $Q_{B}^{max}=300 \text {[ml/min]}$, $Q_{D}^{min}=400 \text {[ml/min]}$, $Q_{D}^{max}=550 \text {[ml/min]}$			
$C^{{PO}_{4}}_{min}=0.70 \text {[mmol/L]}$, $C^{{PO}_{4}}_{max}=0.85 \text {[mmol/L]}$			
*α*=0.05, *β*=300.0, $L_{min}^{UFR}=800 \text {[ml]}$, $L_{max}^{UFR}=1200 \text {[ml]}$, $U_{ufr}^{min}= 0 \text {[ml/min]}$, $U_{ufr}^{max}= 20 \text {[ml/min]}$, *κ*=0.65			
*K*_0_*A*(*urea*)=600[ml/min], $K_{D}^{{PO}_{4}}=220 \text {[ml/min]}$, $K_{r}^{urea}=0 \text {[ml/min]}$, *K*^*ufr*^=12.5[ml/(min·mmHg)]			
$K_{IE}^{urea}=750.0 \text {[ml/min]}$, $K_{IE}^{{PO}_{4}}=350 \text {[ml/min]}$, *V*(0)=48430[ml], *UFR*(*t*_*D*_)=980[ml]			
Input data - patient parameters - as in Table 1			
Constant optimal controls			
Run no.	*Q*_*B*_ [ml/min]	*Q*_*D*_ [ml/min]	*U*_*ufr*_ [ml/min]
1	300	550	4.08
2	400	650	4.08
3	300	504	4.08
Second patient 55 y.o. male			
*t*_*D*_=240[min]			
$L^{urea}_{EC}=5.0 L^{urea}_{IC}=5.5 \text {[mmol/L]}$			
$Q_{B}^{min}=250 \text {[ml/min]}$, $Q_{B}^{max}=300 \text {[ml/min]}$, $Q_{D}^{min}=400 \text {[ml/min]}$, $Q_{D}^{max}=550 \text {[ml/min]}$			
$C^{{PO}_{4}}_{min}=0.70 \text {[mmol/L]}$, $C^{{PO}_{4}}_{max}=0.85 \text {[mmol/L]}$			
*α*=0.05, *β*=300.0, $L_{min}^{UFR}=2500 \text {[ml]}$, $L_{max}^{UFR}=2900 \text {[ml]}$, $U_{ufr}^{min}= 0 \text {[ml/min]}$, $U_{ufr}^{max}= 20 \text {[ml/min]}$, *κ*=0.65			
*K*_0_*A*(*urea*)=600[ml/min], $K_{D}^{{PO}_{4}}=220 \text {[ml/min]}$, *K*^*ufr*^=68.0[ml/(min·mmHg)], $K_{r}^{urea}=0 \text {[ml/min]}$			
$K_{IE}^{urea}=750.0 \text {[ml/min]}$, $K_{IE}^{{PO}_{4}}=350 \text {[ml/min]}$, *V*(0)=43440[ml], *UFR*(*t*_*D*_)=2700[ml]			
Input data - patient parameters - as in Table 1			
Constant optimal controls			
Run no.	*Q*_*B*_ [ml/min]	*Q*_*D*_ [ml/min]	*U*_*ufr*_ [ml/min]
1	300	550	11.25

In the third optimization run the upper boundary $Q^{max}_{D}$ is equal to 550 [ml/min] while the obtained optimal solution of *Q*_*D*_ is equal to 504 [ml/min]. This indicates that there is a conflict between the removal of urea and achieving the desired level of phosphorus concentration at the end of haemodialysis - using optimisation can help to resolve it. On one hand the optimization procedure tries to reach maximum level of *Q*_*D*_ in order to remove urea as much as possible, on the other hand the maximum level $Q_{D}^{max}$ does not guarantee the desired level of phosphorus concentration, eventually the optimization procedure finds the compromise value of *Q*_*D*_. It seems that this type of optimisation problem should be the important one, since the avoidance of low level of phosphorus concentration (and eventually the avoidance of rebound) is the important issue when planning haemodialysis.

As far as optimal values for the control *U*_*ufr*_ are concerned, initially we assumed that $U_{ufr}^{min}= 0$ [ml/min] and $U_{ufr}^{max}= 20$ [ml/min] and the optimization procedure found the following optimal trajectory for *U*_*ufr*_: *U*_*ufr*_=20.00 [ml/min] on the subinterval [0,48), *U*_*ufr*_=4.91 [ml/min] on the subinterval [48,62) and *U*_*ufr*_=0 [ml/min] on the subinterval (62,240] (the time values 48 and 62 follows from the fact that we used piecewise constant approximations for controls and in the approximation the number of subintervals was equal to 10). For this optimization run (the data of the optimization problem are the same as the data for the first patient and run no. 1 in Table 2) values of the other optimal controls were: *Q*_*B*_=300 [ml/min] and *Q*_*D*_=550 [ml/min]. These results show that when we consider an optimization problem with three controls *Q*_*B*_, *Q*_*D*_ and *U*_*ufr*_, then the optimal profile of the rate of ultrafiltration assumes the biggest possible values at the begining of a haemodialysis process and zero values afterwards.

Although ultrafiltration could be modelled, we chose not to do so due to the its decreasing role in routine clinical practice. However, the simulations allows using any profile of ultrafiltration to be modelled in the haemodialysis treatment.

**Fig. 4:**
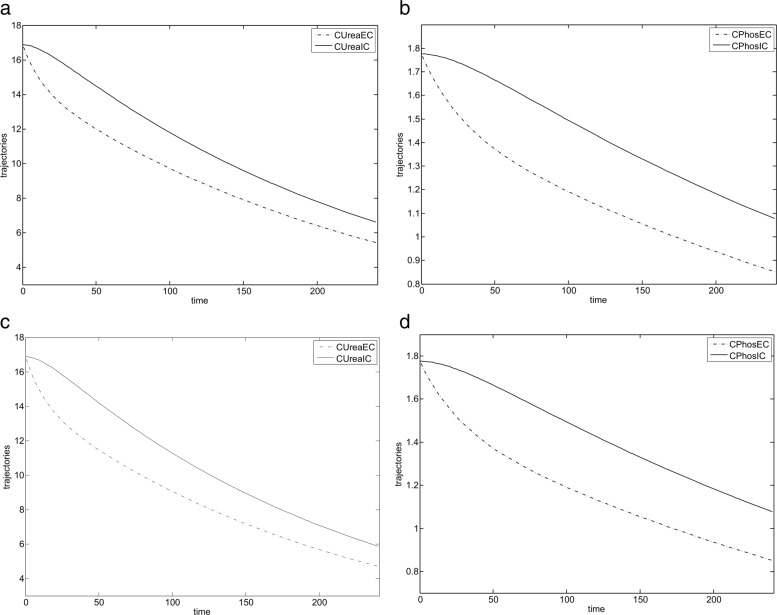
Optimisation results for the first patient. **a** State trajectories for the urea $C_{EC}^{urea}$ and $C_{IC}^{urea}$ for the first run. **b** State trajectories for the phosphorus $C_{IC}^{{PO}_{4}}$ and $C_{EC}^{{PO}_{4}}$ for the first run (**c**) State trajectories for the urea $C_{EC}^{urea}$ and $C_{IC}^{urea}$ for the second run. **d** State trajectories for the phosphorus $C_{IC}^{{PO}_{4}}$ and $C_{EC}^{{PO}_{4}}$ for the second run

For the prescribed haemodialysis parameters and optimal control values, shown in Table 2, the haemodialysis results for the first patient are shown in Fig. 4. Dialysis for the first patient proceeded smoothly and the minimum concentration of phosphorus $C_{min}^{{PO}_{4}}$ in the body was not exceeded.

**Fig. 5:**
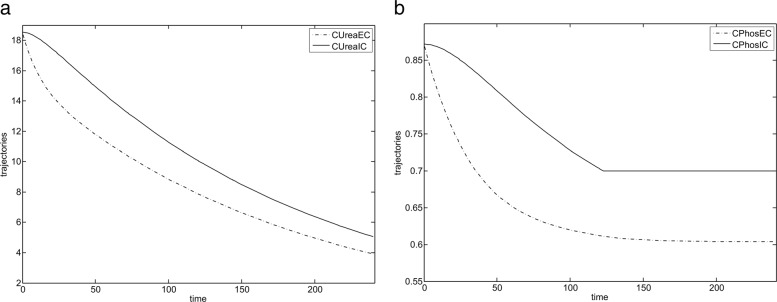
Optimisation results for the second patient. **a** State trajectories for the urea $C_{EC}^{urea}$ and $C_{IC}^{urea}$. **b** State trajectories for the phosphorus $C_{IC}^{{PO}_{4}}$ and $C_{EC}^{{PO}_{4}}$ (rebound of $C_{EC}^{{PO}_{4}}$ can be seen in 120 min of haemodialysis)

During dialysis, second patient experienced a rebound phenomenon due to exceeding the minimum permissible concentration of phosphorus in his body ($C_{min}^{{PO}_{4}}$)— the optimal trajectories obtained for the second patient are shown in Fig. 5.

From these results, one can conclude that in order to achieve the optimal behaviour of haemodialysis (i.e. minimise urea concentration) one could take constant values for *Q*_*B*_ and *Q*_*D*_ (dependent on patient and equipment parameters) – see optimal controls in Table 2.

Figure 5 presents the optimal trajectories representing parameter changes ($C_{EC}^{urea}$ and $C_{IC}^{urea}$) for the second patient. Notice that $C_{EC}^{urea}$ and $C_{IC}^{urea}$ steadily decrease although $C_{IC}^{{PO}_{4}}$ assumes a constant value from some time. Furthermore, Fig. 5 reaffirms our earlier observation that by forcing concentrations of $C_{EC}^{urea}$ and $C_{IC}^{urea}$ to lie below some low values at the end of the haemodialysis process we also guarantee that concentrations of $C_{IC}^{{PO}_{4}}$ and $C_{EC}^{{PO}_{4}}$ steadily decrease.

In the case of a phosphorus concentration for the second patient data one may spot a rebound when a minimum phosphorus level is reached (at the level 0.70 [mmol/L]). In this case a phosphorus extraction mechanisms are activated to preserve a phosphorus balance. It can be seen after 120 min of haemodialysis. From that time the value of $C_{IC}^{{PO}_{4}}$ does not change and the system exhibits a ’sliding mode’ during which it evolves – the details of the system behaviour in this mode are stated in [21].

A rebound process does not appear during urea removal. There is no body mechanism that preserves a minimum level of a toxic urea value. So, a proper balance of urea concentration is close to zero (theoretical value never gained because of nutrition process).

## Discussion

In clinical practice, methods are required to assist nephrologists in the prescription and delivery of safe and effective haemodialysis to patients. There are a number of established physiological models to predict the effect of dialysis on certain solutes, and these allow nephrologists to anticipate the patient response to changes in haemodialysis operating parameters. However, the effects of changing these parameters on urea concentrations, phosphorus concentrations and water volumes are all dependent on each other through differential equations, and it is difficult to predict the patient response for all of these solutes simultaneously. As such, it is also difficult to predict the optimal haemodialysis settings that will result in a different solutes all simultaneously falling within pre-specified limits of safety and efficacy. The use of optimization techniques can cope with problems containing multiple and interdependent differential equations. In our paper, we illustrate the utility of a framing the issue of water and solute changes during haemodialysis as a dynamic optimization problem. We using existing physiological models of solute transport for different solutes, all imbedded within a simulation environment, in a way that results in plausible solutions to this difficult multicriteria optimization problem.

Our paper illustrates the use of this technique. We presented the haemodialysis trajectories for two archetypal patients. The first represents a group that have a rather high level of phosphorus concentration at the start of dialysis with an intention to decrease levels to within, but not below, a clinically acceptable range. The second represents those with starting with a rather low concentration of phosphorus. We set *Q*_*B*_ and *Q*_*D*_ within acceptable ranges, and dialyser clearances to manufacturer specifications, as can be seen in Table 2. For the sake of simplicity, we set $K_{r}^{urea}$ (urea clearance associated with residual renal function) to zero and urea generation during the dialysis to zero. Overall the simulations behaved appropriately and with adequate accuracy. The simulations demonstrated the expected interdependence between ultrafiltration process and the reduction of solute concentrations.

The results of our modelling show that in order to decrease the level of concentrations as much as possible we should use the maximal allowed *Q*_*B*_ and *Q*_*D*_, which is not surprising. Our results also showed that the optimal strategy for solute removal is to maximize *U*_*ufr*_ at the beginning of haemodialysis, which is also not surprising since this is when the greatest solute mass is present in the blood. However, when we need to achieve a certain reduction in urea concentrations but at the same time keep phosphorus concentrations above some prescribed level, these strategies are no longer appropriate and a different set of values for *Q*_*B*_ and *Q*_*D*_ are required. For such patients, the use of dynamic optimization allowed us to estimate these parameters in a reasonably precise manner to achieves a satisfactory reduction in urea concentrations, but at the same time resulting in a reduction in serum phosphate concentration that is not excessive. The use of a IV–compartment model in the simulation allowed detailed modelling of the time-concentration profile of phosphorus, and prediction of the presence, extent, and timing of rebound for given (in particular optimal) *Q*_*B*_ and *Q*_*D*_. Under these conditions, our simulations showed significant rebound of phosphate between 120 and 180 min of haemodialysis.

Our experiment shows that it is feasible to develop a simple interface that uses in the background sophisticated numerical tools (such as numerical integrators for hybrid systems and optimisation procedures for optimisation problems with hybrid systems), with inputs from users that are no different from what is already undertaken in routine clinical care by medical staff while carrying out haemodialysis - routine patients’ parameters, and routine haemodialysis operating parameter. The numerical procedures in OpenModelica can handle this dynamic optimization problem as a simulation with clear and comprehensible results, although it would be better if this functionality in the software was available with sliding modes (unavailable at present). Furthermore, although we used plausible and generic inputs, the model can be individualised to any patient undergoing haemodialysis, with required inputs that can be guessed from cumulative clinical experience or even measured $K_{r}^{urea}$, $C^{{PO}_{4}}_{EC}(0)$, $C^{urea}_{EC}(0)$, $C^{{PO}_{4}}_{min}$, $C^{{PO}_{4}}_{max}$, *V*(0), *G*^*urea*^(0).

Our future research pertaining to the proposed approach will include clinical trials aimed, first of all, at verifying model accuracy, in particular in the case of patients who experience phosphorus rebound during haemodialysis process. Our model contains parameters *α*, *β* related to the rebound effect, these parameters should be estimated on a group of patients by performing the standard procedure for nonlinear regression models ([25]): on a subgroup of patients these parameters are estimated by solving the corresponding nonlinear least squares problem (see [26], [27] for the description of possible numerical procedures to carry out that task) and on the other subgroup of patients the performance of the calibrated model is examined. Note that results in ([7]) indicate that parameters related to rebound do not vary in models used in short and conventional haemodialysis treatments so these parameters could also be estimated individually for patients after performing a trial haemodialysis and then used in the subsequent haemodialyses. Secondly, clinical trials are needed to resolve doubts about negative consequences of performing haemodialysis on the basis of solutions to optimisation problems. To this end stage patients used in clinical trials should be examined with respect to other profiles which are not directly included in optimisation models, such as pH and *HCO*^3^ variation during dialysis ([28]), changes in potassium, sodium, calcium levels and in uremic toxins not routinely controlled in every day practice.

The proposed approach to haemodialysis planning could be applied with other kinetics models which contain parameters that can be measured for individual patients, or could be based on estimates valid across groups of patients (so these estimates could be provided after clinical trials on these groups). Therefore, we intend to extend the presented model by including in it a submodel presenting the nature and the rate of vascular refilling during haemodialysis and ultrafiltration. In [28] such a submodel is presented that links together plasma volume, interstitial volume and the protein concentrations in both compartments. Another extension of our model can incorporate differential equations representing potassium kinetics and constraints related to a desired concentration of potassium at the end of haemodialysis (see, for example, [29]). It should be noticed that potassium concentration can exhibit rebound during haemodialysis ([30]) so the model which includes potassium kinetics is hybrid and our optimization procedure could cope with the problem extended in this way.

All the mentioned directions of planned future research concerning the proposed approach should help to implement a coherent dialysis model suitable for use in real haemodialysis.

## Conclusions

Parameters of the haemodialysis process are routinely determined on the basis of a model of the process and its simulation. Having a model of the process we can go further by employing simulations in optimization of a haemodialysis process. The paper considers several optimization problems associated with haemodialysis and shows results of solving one of them. In that way the paper indicates that applying optimization in haemodialysis is possible and that it can lead to the process improvement.

## Data Availability

Data sets generated and/or analysed during the current study are not publicly available because they are part of the medical records of the patients involved. Modelica models can be made available by the corresponding author upon reasonable request.

## Abbreviations

CVD: Cardiovascular disease
DOML: Dynamic optimization modeling language
HD: Haemodialysis
TBW: Total body water

## References

[1] Natoli J, Boer R, Nathanson B, Miller R, Chiroli S, Goodman W, Belozeroff V (2013). Is there an association between elevated or low serum levels of phosphorus, parathyroid hormone, and calcium and mortality in patients with end stage renal disease?. BMC Nephrol.

[2] Tentori F, Blayney M, Albert J, Gillespie B, Kerr P, Bommer J, Young E, Akizawa T, Akiba T, Pisoni R, Robinson B, Port F (2008). Mortality risk for dialysis patients with different levels of serum calcium, phosphorus, and pth: the dialysis outcomes and practice patterns study (dopps). Am J Kidney Dis.

[3] Copland M, Komenda P, Weinhandl E, McCullough P, Morfin J (2016). Intensive hemodialysis, mineral and bone disorder, and phosphate binder use. Am J Kidney Dis.

[4] KDIGO (2017). Kdigo 2017 clinical practice guideline update for the diagnosis, evaluation, prevention, and treatment of chronic kidney disease-mineral and bone disorder (ckd-mbd). Kidney Int Suppl.

[5] Minutolo R, Bellizzi V, Cioffi M, Iodice C, Giannattasio P, Andreucci M, Terracciano V, Di Iorio B, Conte G, Nicola LD (2002). Postdialytic rebound of serum phosphorus: pathogenetic and clinical insights. J Am Soc Nephrol.

[6] Daugirdas J (2015). Removal of phosphorus by hemodialysis. Semin Dial.

[7] Agar B, Akonur A, Ying-Cheng L (2011). Kinetic model of phosphorus mobilization during and after short and conventional hemodialysis. Clin J Am Soc Nephrol.

[8] Borah MF, Schoenfeld PY, Gotch FA (1978). Nitrogen balance during intermittent dialysis therapy of uraemia. Kidney Int.

[9] Pohlmeier R, Vienken J (2001). Phosphate removal and hemodialysis conditions. Kidney Int.

[10] Ratanarat R., Brendolan A., Volker G. (2005). Phosphate kinetics during different dialysis modalities. Blood Purif.

[11] Spalding E, Chamney P, Farrington K (2002). Phosphate kinetics during hemodialysis: Evidence for biphasic regulation. Kidney Int.

[12] Gotch F, Panlilio F, Sergeyeva O, Rosales L, Folden T, Kaysen G, Levin N (2003). A kinetic model of inorganic phosphorus mass balance in hemodialysis therapy. Blood Purif.

[13] Leypoldt J, Agar B, Culleton B (2014). Simplified phosphorus kinetic modeling: predicting changes in predialysis serum phosphorus concentration after altering the hemodialysis prescription. Nephrol Dial Transplant.

[14] Lopot F, Filpot F (1990). Evolution of mathematical methods for the assessement of dialysis adequacy. Urea Kinetic Modelling vol. 4.

[15] Watson P, Watson I, Batt R (1980). Total body water volumes for adult males and females estimated from simple anthropometric measurements. Am J Clin Nutr.

[16] Mieczkowski M, Lesniak K, Matuszkiewicz-Rowinska J (2012). Potassium modeling during hemodialysis in patients with end-stage renal disease - a pilot study. Nefrol Dial Pol.

[17] Gotch FA, Kotanko P, Handelman G (2007). A kinetic model of calcium mass balance during dialysis therapy. Blood Purif.

[18] Branicky MS, Borkar VS, Mitter SK (1998). A unified framework for hybrid control: model and optimal control theory. IEEE Trans Autom Control.

[19] van der Schaft A, Schumacher H (1989). An Introduction to Hybrid Dynamical Systems.

[20] Pytlak R, Suski D (2017). On solving hybrid optimal control problems with higher index daes. Optim Methods Softw.

[21] Pytlak R., Suski D., Tarnawski T., Awrejcewicz J. (2018). Optimal control of hybrid systems with sliding modes. Springer Proceedings in Mathematics and Statistics.

[22] Pytlak R (1999). Numerical Methods for Optimal Control Problems with State Constraints. Lecture Notes in Mathematics 1707.

[23] Hairer E, Wanner G (1996). Solving Ordinary Differential Equations II.

[24] Modelica. A Unified Object-Oriented Language for Systems Modeling: Modelica Press; 2012. https://www.modelica.org/documents.

[25] McCoy T, Castro V, Cagan A, Roberson A, Perlis R (2017). Validation of a risk stratification tool for fall related injury in a state wide cohort. BMJ Open.

[26] Nocedal J, Wright S (2006). Numerical Optimization.

[27] Pytlak R, Tarnawski T. Idos - (also) a web based tool for calibrating modelica models. In: Proceedings of the 10th International Modelica Conference. Lund: 2014. p. 1095–1104. 10.3384/ECP140961095.

[28] Harris D, Yull E, Chester D (1995). Correcting acidosis in hemodialysis: Effect on phosphate clearance and calcification risk. J Am Soc Nephrol.

[29] Ciandrini A, Severi S, Grandi F, Mura C (2009). Model-based analysis of potassium removal during haemodialysis. Artif Organs.

[30] Ursio M, Donati G (2017). Mathematical model of potassium profiling in chronic dialysis. Contrib Nephrol.

